# COVID-19-Associated Superior Mesenteric Artery Thrombosis and Acute Intestinal Ischemia

**DOI:** 10.7759/cureus.27722

**Published:** 2022-08-05

**Authors:** Fernando D Segovia, Sarah Ream, The Dang, Bhanu T Chaganti, Andrew J Ortega, Seunghong Rhee, Jorge C Borges

**Affiliations:** 1 Internal Medicine, Texas Tech University Health Sciences Center, El Paso, USA; 2 Internal Medicine, Paul L. Foster School of Medicine, Texas Tech University Health Sciences Center, El Paso, USA; 3 Cardiovascular Medicine, Texas Tech University Health Sciences Center, El Paso, USA; 4 Radiology, Texas Tech University Health Sciences Center, El Paso, USA

**Keywords:** covid-19 associated coagulopathy, acute gi pathology, sars-cov-2 (severe acute respiratory syndrome coronavirus -2), bowel, vascular, gastrointestinal, thrombus, mesenteric, ischemia, covid-19

## Abstract

The prothrombotic nature of severe acute respiratory syndrome coronavirus 2 (SARS-CoV-2) has been well-established since the start of the global coronavirus disease 2019 (COVID-19) pandemic. Mesenteric artery thrombosis and acute mesenteric ischemia are, on their own, rare occurrences and often present with fatal gastrointestinal (GI) pathologies requiring quick identification and intervention by the clinician to improve clinical outcomes. SARS-CoV-2 infection can present with acute GI pathologies and warrants further investigation regarding anticoagulation therapy in COVID-19 positive patients. We report on a 64-year-old woman infected with SARS-CoV-2 who presented with superior mesenteric artery thrombosis and acute intestinal ischemia.

## Introduction

Infection with severe acute respiratory syndrome coronavirus 2 (SARS-CoV-2) causing coronavirus disease 2019 (COVID-19) has been increasingly associated with coagulopathy and thrombotic complications. Although pulmonary presentations of the disease have predominated, extrapulmonary complications have also been reported in individuals with confirmed COVID-19 [1,2]. Acute mesenteric ischemia (AMI) is a less common thrombotic complication, being described in only a few case reports [3], but with high morbidity and mortality [1,2,4]. This report describes a patient affected by COVID-19 presenting as superior mesenteric artery (SMA) thrombosis and acute intestinal ischemia.

## Case presentation

**Figure 1:**
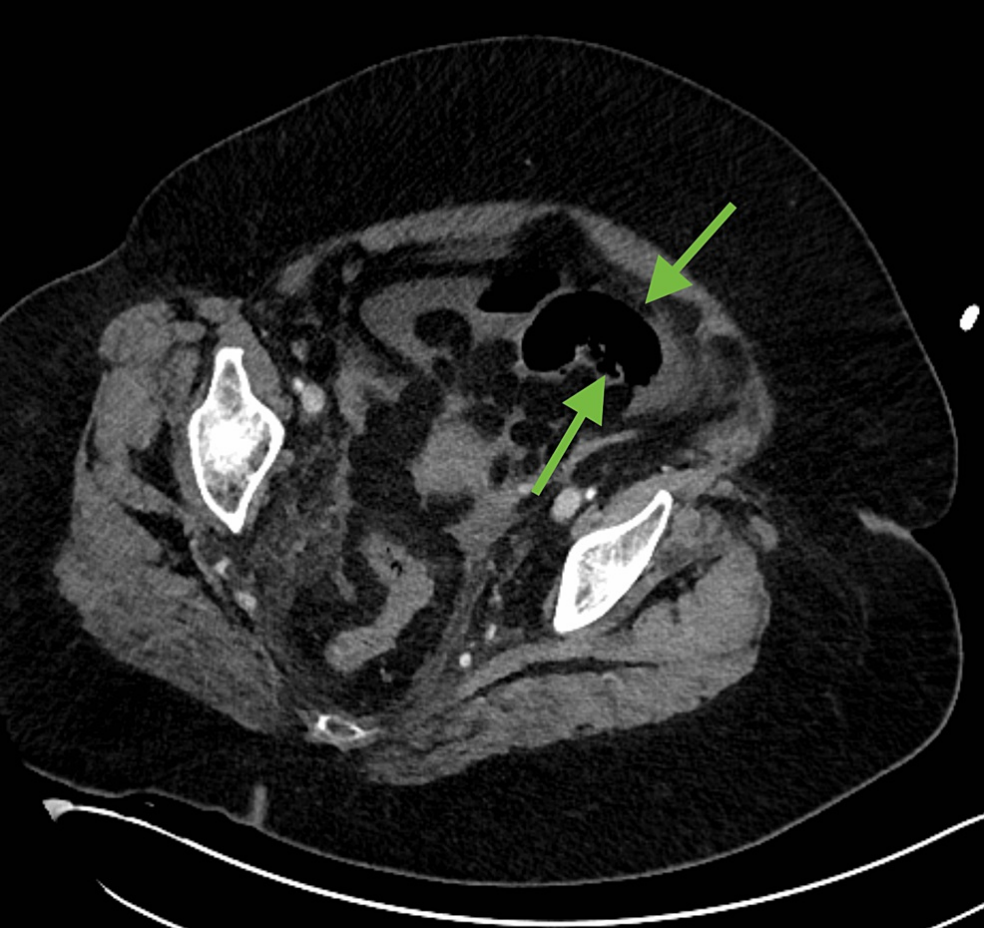
Axial soft tissue window does not allow for adequate visualization of bowel wall pneumatosis (green arrows).

**Figure 2:**
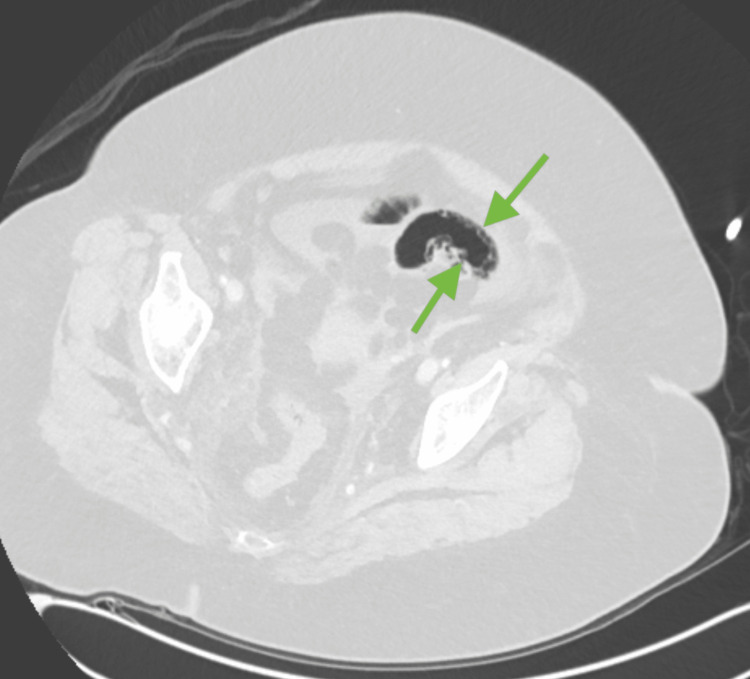
Axial lung window allows for visualization of bowel wall pneumatosis (green arrows).

**Figure 3:**
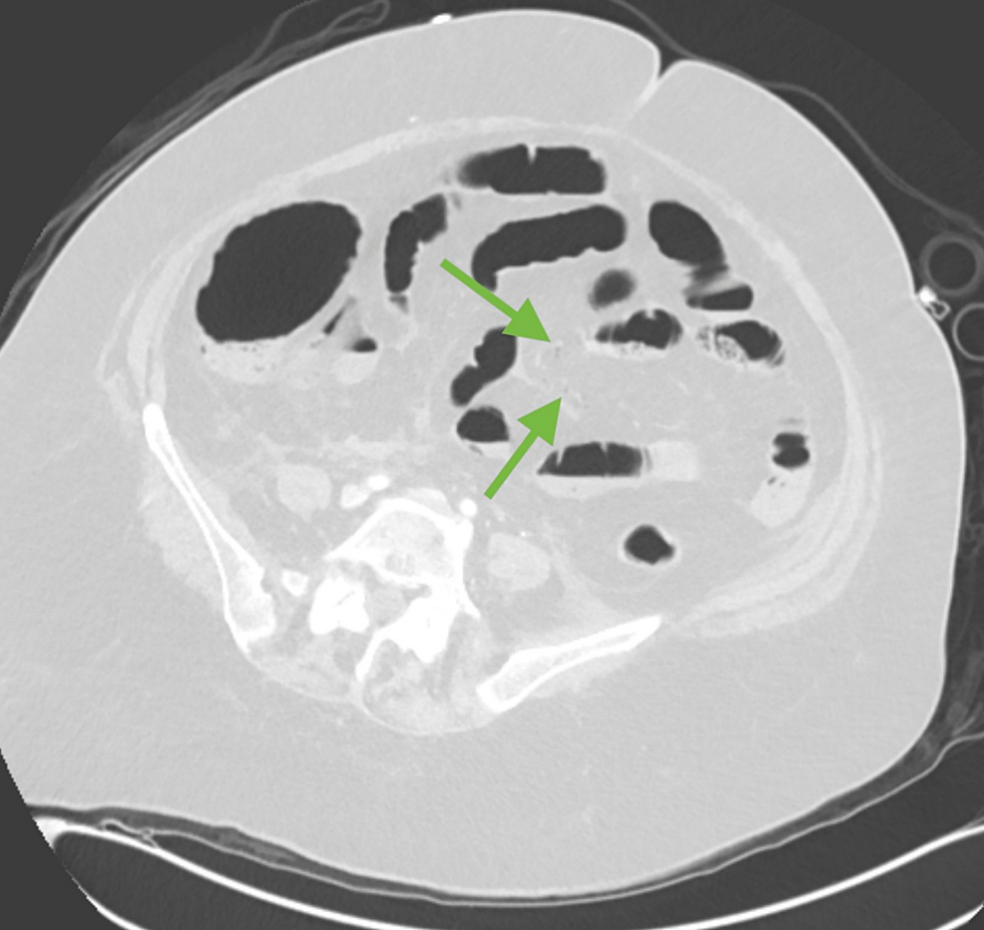
Axial lung window demonstrating very subtle mesenteric vein air (green arrows).

**Figure 4:**
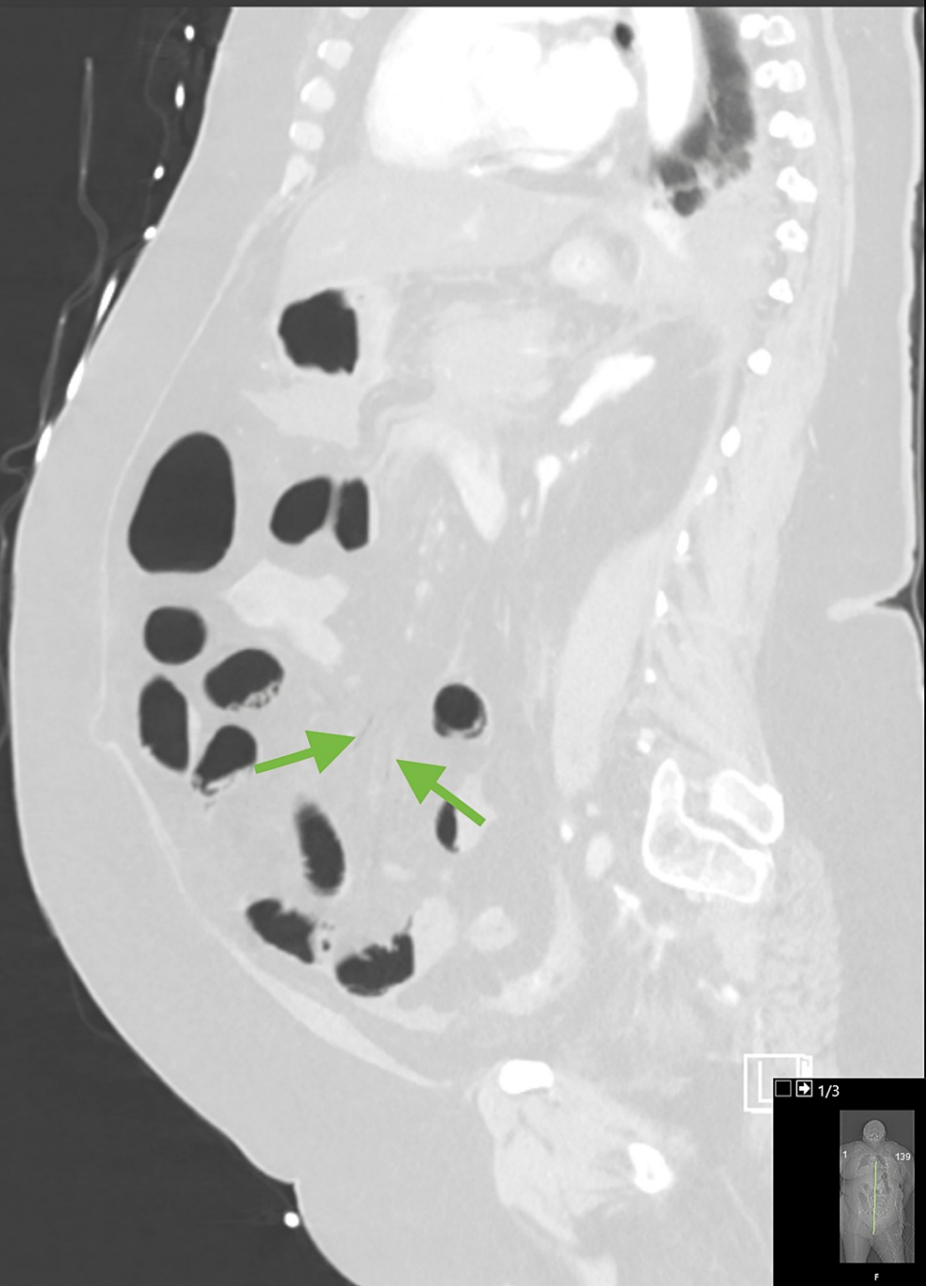
Saggital reformat in lung window again demonstrating very subtle mesenteric vein air (green arrows).

A 64-year-old female with a past medical history of hypertension and diabetes mellitus presented to the emergency department after experiencing two days of constipation, abdominal pain, and distention. While waiting in triage, the patient collapsed and became unresponsive. She was found to be hypotensive with a Glasgow Coma Scale (GCS) of 5 and was quickly taken to the trauma bay, intubated, and started on vasopressors. Laboratory workup was significant for lactic acidemia of 9.6 mmol/L, a predominantly neutrophilic leukocytosis of 19.37 x 10^3 cells/L, elevated D-dimer of >20 ug/mL, and SARS-CoV-2 detected on a BioFire® respiratory panel (BioFire Diagnostics, Salt Lake City, Utah, United States). EKG showed sinus tachycardia and there was a slight troponin elevation, compatible with type II myocardial infarction (MI). A CT abdomen and pelvis with contrast was significant for diffuse pneumatosis in the small bowel loops in left lower quadrant and pelvis (Figures 1, 2) as well as diffuse decreased caliber of the celiac axis and superior mesenteric artery with air pockets in the mesenteric vessels of the left lower quadrant (Figures 3, 4).

These findings were highly suggestive for non-occlusive bowel ischemia and the patient was taken for an urgent exploratory laparotomy. At that time, it was discovered that the patient had a large area of ischemia in the distribution of the SMA, which was removed, along with significant ischemia to the large bowel prompting total colectomy; the patient was left with a remaining estimated 150 cm of viable small bowel at closure. An ABTHERA™ wound vacuum-assisted closure (VAC) (Acelity L.P. Inc., San Antonio, Texas, United States) was placed and the patient was taken back to the ICU for continued monitoring and care. Over the next two postoperative days on high dose vasopressors, the patient continued to deteriorate with progressive multiorgan failure. The patient was taken for a second urgent exploratory laparotomy, which was significant for a 1 cm area of necrosis on the anterior aspect of the rectal stump. Consequently, the rectal stump was resected, effectively removing the area of ischemia; however, the patient continued to decline clinically. Nine days after admission to the hospital, the patient’s family decided on comfort care measures only, after which the patient rapidly expired.

## Discussion

It has been suggested that the coagulopathy provoked by SARS-CoV-2 is due to microcirculatory changes. One hypothesis proposes that viral replication causes inflammatory cells to infiltrate the endothelium leading to endothelial apoptosis and subsequent microvascular prothrombotic events [5]. In addition, SARS-CoV-2 has been shown to act on angiotensin-converting enzyme 2 receptors in the lungs, which are also found in vascular endothelium and in enterocytes of the small intestine, supporting SARS-CoV-2 microvascular thrombotic effects on small bowel [6]. Pulmonary embolism presentation accounts for the majority of COVID-19 related coagulopathies; however, there are reported cases including venous thromboembolism, arterial thrombosis, MI, stroke, and microvascular thrombosis [5].

Infection with SARS-CoV-2 occurs by aerosol droplet inhalation and is primarily characterized by respiratory symptoms. GI manifestations of COVID-19 such as nausea, vomiting, diarrhea, and abdominal pain have been well-documented; however, the true prevalence of GI symptoms among COVID-19 positive patients are unknown, ranging from less than 10% up to 70% in different reports [3,7]. While AMI is rare with an overall incidence less than 1%, AMI in the setting of COVID-19 warrants a high index of suspicion to avoid detrimental, possibly fatal, complications [8]. The recent Medically Ill Hospitalized Patients for COVID-19 Thrombosis Extended Prophylaxis With Rivaroxaban Therapy (MICHELLE) randomized, controlled trial suggests improved clinical outcomes with extended use of rivaroxaban anticoagulation in high-risk patients following discharge from the hospital, supporting thromboprophylaxis for patients at increased risk of thrombotic events [9].

## Conclusions

With the SARS-CoV-2 virus still creating a significant burden on the healthcare, various unforeseen pathological manifestations continue to be described. Thromboembolic presentations of the virus, such as AMI, present significant clinical challenges to physicians due to its unpredictable and catastrophic nature. Early recognition of AMI and identifying those at highest risk are important for prompt clinical diagnosis and treatment, which may lead to better clinical outcomes. Future investigation regarding prophylactic anticoagulation therapy in COVID-19 positive patients is warranted considering individual patient risk and the high morbidity and mortality associated with AMI.

## References

[1] Keshavarz P, Rafiee F, Kavandi H, Goudarzi S, Heidari F, Gholamrezanezhad A (2021). Ischemic gastrointestinal complications of COVID-19: a systematic review on imaging presentation. Clin Imaging.

[2] Kaafarani HM, El Moheb M, Hwabejire JO (2020). Gastrointestinal complications in critically ill patients with COVID-19. Ann Surg.

[3] Serban D, Tribus LC, Vancea G (2021). Acute mesenteric ischemia in COVID-19 patients. J Clin Med.

[4] Ojha V, Mani A, Mukherjee A, Kumar S, Jagia P (2022). Mesenteric ischemia in patients with COVID-19: an updated systematic review of abdominal CT findings in 75 patients. Abdom Radiol (NY).

[5] Abou-Ismail MY, Diamond A, Kapoor S, Arafah Y, Nayak L (2020). The hypercoagulable state in COVID-19: incidence, pathophysiology, and management. Thromb Res.

[6] Al Mahruqi G, Stephen E, Abdelhedy I, Al Wahaibi K (2021). Our early experience with mesenteric ischemia in COVID-19 positive patients. Ann Vasc Surg.

[7] Groff A, Kavanaugh M, Ramgobin D, McClafferty B, Aggarwal CS, Golamari R, Jain R (2021). Gastrointestinal manifestations of COVID-19: a review of what we know. Ochsner J.

[8] Bala M, Kashuk J, Moore EE (2017). Acute mesenteric ischemia: guidelines of the World Society of Emergency Surgery. World J Emerg Surg.

[9] Ramacciotti E, Barile Agati L, Calderaro D (2022). Rivaroxaban versus no anticoagulation for post-discharge thromboprophylaxis after hospitalisation for COVID-19 (MICHELLE): an open-label, multicentre, randomised, controlled trial. Lancet.

